# Supplementary figures and images for: Genetic Diversity and Evaluation of Agro-Morphological Traits in Lettuce Core Collection
Source: Plants (Basel). 2024 Dec 19;13(24):3552. doi: 10.3390/plants13243552 (PMC11679554; doi:10.3390/plants13243552)

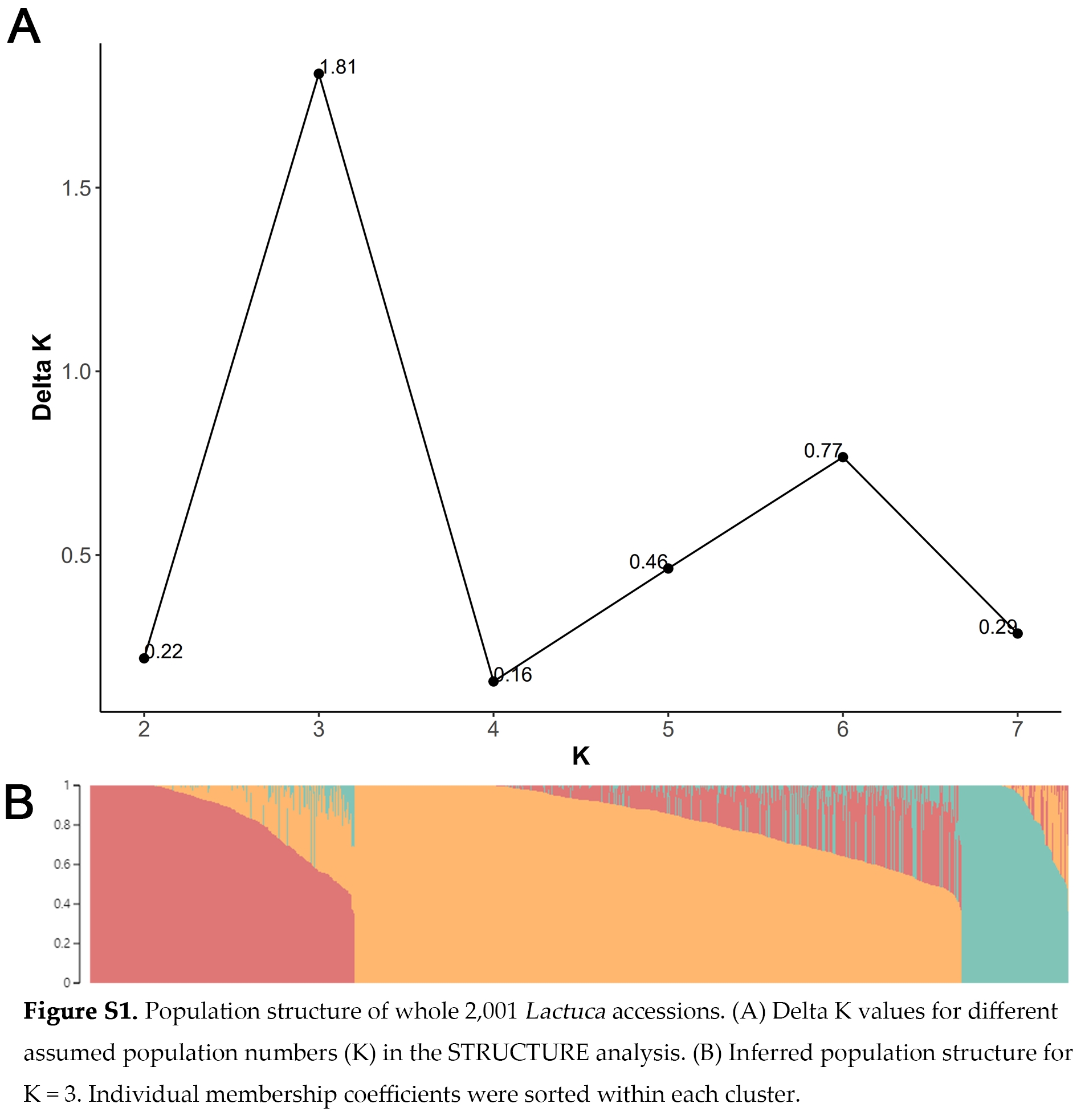

Supplement: Supplementary file 1 [file plants-13-03552-s001.zip › Figure S1.jpg]
